# Doppler sonography of the umbilical and uterine arteries blood flow of pregnant Saanen goats

**DOI:** 10.1590/1984-3143-AR2022-0014

**Published:** 2022-10-10

**Authors:** Priscila Del’Aguila-Silva, Fabiana Cirino dos Santos, Victor José Correia dos Santos, Ricardo Andres Ramirez Uscategui, Luciana Cristina Padilha-Nakaghi, Renata Sitta Gomes Mariano, Mariana Garcia Kako Rodriguez, Lizandra Amoroso, Wilter Ricardo Russiano Vicente, Marcus Antônio Rossi Feliciano

**Affiliations:** 1 Departamento de Patologia, Reprodução e Saúde Única, Faculdade de Ciências Agrárias e Veterinárias, Universidade Estadual Paulista “Júlio de Mesquita Filho”, Jaboticabal, SP, Brasil; 2 Grupo de investigación INCA-CES, Facultad de Medicina Veterinaria y Zootecnia, Universidad CES, Medellín, Colombia; 3 Departamento de Medicina Veterinária, Faculdade de Zootecnia e Engenharia de Alimentos, Universidade de São Paulo, Pirassununga, SP, Brasil

**Keywords:** ultrasonography, Saanen, maternal-fetal vascularization

## Abstract

The objective was to evaluate the blood flow of the uterine artery (UA) and umbilical artery (UMB) in the physiological pregnancy of goats by means of Doppler throughout the gestational period. Twenty-five Saanen goats weighing 55 ± 10 kg and aged between 2 and 5 were evaluated weekly, from the 21^st^ until the 143^rd^ day of gestation, and daily from that period until parturition. Values for peak systolic velocity (PSV), end diastolic velocity (EDV) and resistance (RI) and pulsatility (PI) indices of the uterine and umbilical arteries were determined. The values obtained were correlated with gestational age by Spearman's test, tested for adjustment to regression models and compared with the number of fetuses by ANOVA. The umbilical cord was first visualized at 28 days. Of the variables evaluated, RIUMB and PIUMB correlated with gestational age (p<0.001; and 0.046; respectively) and RIUMB had a low negative correlation with the number of fetuses per pregnancy (p = 0.003; r- Spearman = - 0.218). PSVUMB and EDVUMB values did not correlate with gestational age (p=0.737 and 0.768, respectively), but there was a decrease in the mean values throughout pregnancy (PSVUMB= 0.07; 0.31 and EDVUMB= 0.01; 0.06) as well as the change in the flow pattern of the spectral trace. The mean values of the uterine artery dopplervelocimetric variables PSVUT, EDVUT, PIUT and RIUT did not correlate with gestational age (p= 0.324; 0.372, 0.143; 0.13; respectively). It is expected that the results obtained will contribute to a broader understanding of the hemodynamic changes resulting from pregnancy in goats.

## Introduction

The Doppler technique, in association with the B-mode, provides real-time information on the vascular architecture and hemodynamic aspects of vessels in various organs (Carvalho et al., 2008). In obstetric evaluation, this method has been useful to assess the health status of the mother and fetus, in addition to aiding in the prognosis, degree and duration in cases of uterine torsion, metritis, placental retention, mastitis and dystocic births (Heppelmann et al., 2013; Santos et al., 2015).

During pregnancy, the use of color and pulsed Doppler provides qualitative and quantitative vascular information about maternal and fetal hemodynamic characteristics, such as velocity, circulation and blood type (arterial or venous), which has important clinical gynecological implications for human and animal species (Kumar et al., 2015). The measured vascular perfusion is based on the evaluation of resistance, pulsativity and systolic and diastolic velocities, in addition to the blood flow volume (Giannico et al., 2015).

Evaluating the hemodynamic characteristics of umbilical vessels in pregnant Beetal goats, Kumar et al. (2015) found that peak systolic velocity (PSV) increased significantly from day 39 to 67 and thereafter between 98 and 120 days of gestation (p < 0.05), but there was no significant increase or decrease in end-diastolic velocity (EDV).

In sheep, Beltrame et al. (2017) found that the Doppler flowmetric parameters and the diameter of the uterine artery varied significantly throughout pregnancy. The resistance index (RI) and the systole/diastole (S/D) ratio decreased after the initial stage of pregnancy and remained from the middle to the end of pregnancy without major variations. The pulsatility index (PI) was inversely proportional to the gestational age.

The aim of this study was to obtain the uterine and umbilical artery Doppler flowmetric indices in pregnant Saanen goats, throughout pregnancy, in order to contribute to increase the knowledge on the vascular physiology of pregnancy in this species and its correlation with gestational age.

## Methods

A prospective longitudinal study was carried out with 25 Saanen, multiparous, adult goats (2-5 years) with a body weight of 55 ± 10 kg, healthy by physical, obstetrical examinations and history with no reported comorbidities. This study was approved by the Animal Welfare and Ethics Committee of Universidade Estadual Paulista protocol nº 010229/17.

The goats underwent a short estrus synchronization protocol that consisted of: placement of an intravaginal device containing 0.33 g of progesterone (Eazi breed CIDR®, Pfizer, New Zealand) on day 0 (random day of the cycle); administration of 37.5 µg of cloprostenol (Sincrocio®, Ourofino, Cravinhos, SP, Brazil) and 300 IU of equine chorionic gonadotropin (Novormon® Coopers MSD, Brazil) on the 5th day; and removal of the implant on the 6th day. After 24 hours (7th day), the females were allocated with a sire for 24 hours, so that the exact day of gestation could be ensured.

### Ultrasound evaluation

The animals were kept in a quadrupedal position and the exams were performed on the MyLab-30-VET device (Esaote, Genova, Italy). At 21 days after copulation, gestation was assessed using the transrectal route. Once pregnancy was confirmed, weekly evaluations were performed until 56 days of gestation, using the same transrectal approach, with a specific 7.5 MHz transducer, with removal of feces from the rectum and using ultrasound gel for lubrication and to favor contact and image quality. The evaluation of the uterine artery was performed using the transrectal approach throughout the gestational period.

From the 63^rd^ gestational day until the end of pregnancy, the evaluations of the umbilical cord were carried out through the transabdominal route, and at this stage of pregnancy the uterus was already displaced towards the abdominal wall, due to the increase in the size and weight of the maternal-fetal structures. A 7.5 MHz linear transducer was used, with previous wide trichotomy of the inguinal and abdominal regions, and ultrasound gel was applied to the abdomen to favor the contact and quality of image. The transducer was positioned in the animal's right abdominal region, cranial to the udder, and then abdominal scan was performed sweeping the abdomen from right to left until the umbilical cord was visible (Kähn, 1994).

From day 143 of gestation, the animals were evaluated daily until parturition. In this last week's assessment, the variables started to be related to the hours before parturition (HBP) and no longer to the gestational days. The time of all assessments was recorded as well as the time of parturition and the relationship between these was calculated in a regressive way.

In all exams, only one conceptus of each female was evaluated, regardless of whether the pregnancy was simple or multiple, but the number of conceptuses visualized was noted, because, as the pregnancy progresses, the evaluation of two or more fetuses becomes complex, it is difficult to guarantee that the structures of the same concept were not evaluated more than once.

### Doppler ultrasonography

The umbilical arteries were evaluated at an intermediate site in the free-floating umbilical cord, as described by Serin et al. (2010). To assess the uterine artery, the urinary vesicle was first located, and the adjacent flow was measured immediately craniolaterally as described by Elmetwally (2016). After identifying the vessels of interest using color mapping, a sample volume was placed in the central portion of the vascular structures, and then pulsed Doppler (spectral) was activated.

Tracings of the region of interest from the vessel were obtained with at least three subsequent waves. After correcting the insonation angle (as close to 0 degrees as possible; maximum of 60º) and with artifact-free tracing, the image was frozen and wave morphology analysis was performed, automatically obtaining the peak systolic velocity (PSV), end diastolic velocity (EDV), mean velocity (MV) resistance (RI [PSV-EDV/PSV]) and pulsatility indexes (PI [PSV-EDV/MV]) (Feliciano et al., 2012). In addition to the quantitative assessment of the Dopplervelocimetric variables, the aspects of spectral tracings were described qualitatively.

### Statistical analysis

It was carried out using the R-project software (R® foundation for statistical computing, Austria). Spearman's correlation analysis was used to determine the relationship between the variables studied, the gestational days and the number of fetuses. When correlation resulted significant, adjustments of the variables and gestational days to regression models (linear, quadratic and cubic) were then tested, and the one that resulted in the highest R^2^ was reported. Subsequently, a regression of best subsets was performed to determine associations that would allow the estimation of gestational age. Additionally, variables were compared over time between the number of fetuses per pregnancy by analysis of variance with repeated measures over time and Tukey's post-test. The significance level was set at 5% (p<0.05) for all of the tests and the results presented as the mean ± SD (standard deviation).

## Results

At 21 days after mating, it was possible to confirm the pregnancy, by the visualization of anechogenic content inside the uterine lumen, suggesting the presence of fluid and characterizing the embryonic vesicle. Of the 25 evaluated animals, five developed single pregnancies, 15 twin pregnancies, four triplets and one fivefold gestation, resulting in the birth of 52 kids with a mean weight of 3.21 ± 0.80 kg.

### Umbilical artery

The umbilical cord was first detected at 28 days of gestation. From day 63 on, only 15 animals could be evaluated and this number gradually decreased each week due to the difficulty of visualizing the umbilical cord, until day 133, the last day where the vascularization of the umbilical artery could be assessed in our examinations.

**Figure 1:**
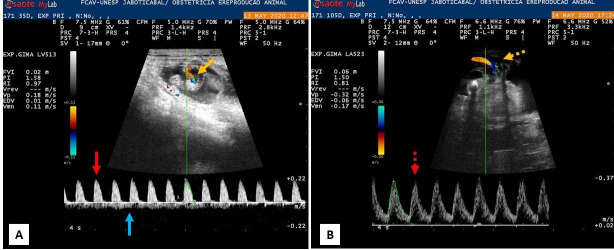
Color and spectral Doppler ultrasound images of the umbilical artery in gestation of goats in embryonic and fetal stages. A: Spectral Doppler tracing of a goat embryo at 35 days of gestation. It is possible to visualize the umbilical cord in longitudinal section (continuous yellow arrow) with the use of color Doppler and the serrated wave pattern (continuous red arrow) with the flow of the umbilical vein below the baseline (solid blue arrow). B: Spectral Doppler tracing (dotted red arrow) at 105 days of gestation, it is possible to visualize the umbilical cord in a longitudinal section (dashed yellow arrow).

Throughout pregnancy, the profile of spectral tracing of the umbilical flow varied. In the first third of pregnancy: monophasic pattern of high resistance, with fine systolic peak and above the baseline, without the presence of the diastolic flow, together with a flat flow below the baseline (umbilical vein) (Figure 1A); from days 77-84, the wave pattern showed intermediate resistance, presence of the diastolic component and absence of the umbilical vein flow below the baseline (Figure 1B).

The RIUMB and PIUMB were correlated negatively and positively with gestational age (p < 0.001; and P = 0.046; respectively), however presented low coefficients of determination, even though combined in the same predictive formula as shown in Table 1. Values of these variables throughout pregnancy are described in Table 2.

**Table 1:** Values for umbilical artery Doppler flowmetric indices and equations for determining gestational age in Saanen goats.

**Variable**	**VI**	**Predictive equation**	**R^2^%**	**r-Spearman**
RIUMB	28-133	GA = 263.8 – 210.4 x RIUMB	33.7	-0.584
PIUMB	28-133	GA = 1283 – 2120 x PIUMB + 1189 IPUMB^2^ – 214.6 x IPUMB^3^	26.4	0.145
RIUMB + PIUMB	28-133	GA = 247.398 – 287.013 RIUMB + 53.9688 PIUMB	52.2	-

VI: visualization interval; GA: gestational age; R^2^: determination coefficient; r-Spearman: correlation coefficient; RIUMB: resistance index in umbilical artery; PIUMB: pulsatility index on umbilical artery.

**Table 2:** Umbilical artery Doppler flowmetric parameters in Saanen goats obtained by means of non-invasive spectral Doppler ultrasonography during the gestational period.

**Variable**	**GA**	**N**	**Mean**	**SD**	**CI 95% IL**	**CI 95% SL**
RIUMB	28	13	0.96	0.07	0.92	1.00
	35	21	0.98	0.03	0.96	0.99
	42	23	0.99	0.03	0.97	1.00
	49	24	0.98	0.02	0.98	0.99
	56	22	0.97	0.03	0.96	0.98
	63	15	0.98	0.04	0.96	1.00
	70	11	0.96	0.05	0.94	0.99
	77	11	0.95	0.03	0.93	0.97
	84	10	0.95	0.04	0.92	0.97
	91	8	0.92	0.04	0.89	0.95
	98	7	0.92	0.09	0.86	0.99
	105	9	0.83	0.06	0.79	0.86
	112	4	0.88	0.09	0.79	0.96
	119	6	0.83	0.19	0.68	0.99
	126	4	0.77	0.13	0.64	0.90
	133	2	0.85	0.16	0.63	1.07
PIUMB	28	13	1.53	0.18	1.43	1.63
	35	21	1.59	0.13	1.54	1.65
	42	23	1.54	0.14	1.48	1.59
	49	24	1.56	0.14	1.50	1.61
	56	22	1.61	0.12	1.56	1.66
	63	15	1.79	0.23	1.67	1.90
	70	11	1.92	0.29	1.75	2.09
	77	11	1.81	0.19	1.69	1.92
	84	10	1.93	0.17	1.82	2.04
	91	8	1.81	0.21	1.66	1.96
	98	7	1.75	0.32	1.51	1.98
	105	9	1.61	0.26	1.44	1.78
	112	4	1.69	0.32	1.37	2.01
	119	6	1.54	0.54	1.11	1.97
	126	4	1.23	0.24	0.99	1.46
	133	2	1.61	0.31	1.18	2.04

GA: gestational age; N: number of assessed animals; Mean: average of the values obtained; SD: standard deviation; CI: confidence interval; IL: inferior limit; SL: superior limit; RIUMB: umbilical artery resistivity index; PIUMB: umbilical artery pulsatility index.

Comparing the values of the Doppler velocimetric indices with the number of fetuses per pregnancy, the RIUMB obtained was higher (p = 0.016) in pregnancies with 1 and 2 fetuses than in pregnancies with 3 and 5 fetuses. When these variables were correlated with the number of fetuses, the RIUMB showed a low negative correlation (p = 0.003; r-Spearman = -0.218), indicating that the greater the number of fetuses, the lower the RIUMB value.

Variables PSVUMB and EDVUMB did not correlate with gestational age (p=0.737 and 0.768; respectively), however, there was a decrease in the mean values of these variables when comparing the first and last assessment: PSVUMB= 0.07; 0.31 and EDVUMB = 0.01; 0.06 performed at 28 and 133 days of gestation, respectively, as well as the change in the spectral tracing flow pattern (Figure 1AB).

### Uterine artery

**Figure 2:**
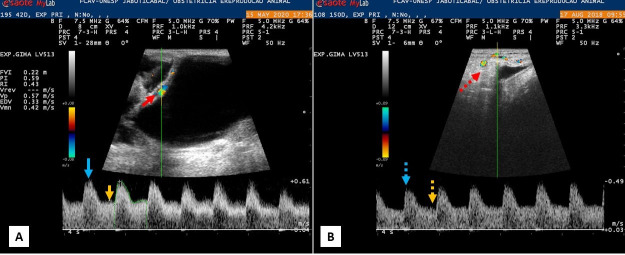
Color and spectral Doppler ultrasound images of the uterine artery in gestation of goats in embryonic and fetal stages. A: Uterine artery flow (solid red arrow) at 42 days of gestation, SPV (solid blue arrow) and EDV (solid yellow arrow). B: Uterine artery flow (dashed red arrow) at 150 days of gestation. Spectral tracing shows SPV (dashed blue arrow) and EDV (dashed yellow arrow).

**Figure 3:**
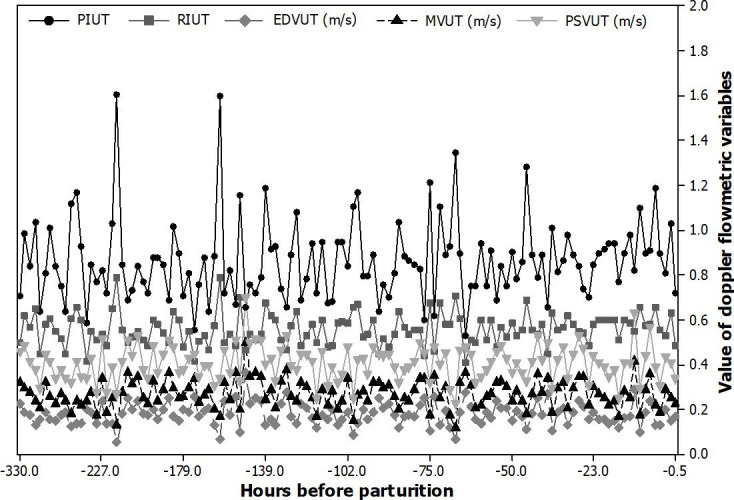
Plot of mean ± standard error of uterine artery pulsativity index (PIUT), resistance index (RIUT), end-diastolic velocity (EDVUT - m/s), mean velocity (MVUT – m/s), and peak systolic velocity (PSVUT - m/s) of Saanen goats in the peripartum period (hours before parturition).

The uterine artery could be accessed throughout the entire pregnancy by the transrectal route in a practical and applicable way (Figure 2). The Doppler velocimetric variables related to the uterine artery did not show a significant increase or decrease during the gestational period evaluated and none of these variables correlated with the time of parturition (Figure 3).

The mean values of the uterine artery Doppler velocimetric variables PSVUT, EDVUT, PIUT and RIUT did not correlate with gestational age (p= 0.324; 0.372, 0.143; 0.13; respectively). When comparing the first and last assessments, PSVUMB= -0.10; -0.35 and EDVUMB= -0.04; -0.015 performed at 21 and 150 days of gestation, respectively, while the mean values of PI and RI are described in Table 3. When these variables were correlated with the number of fetuses per pregnancy, PSVUT and MVUT (mean velocity of uterine artery) were negatively correlated with the number of fetuses (p= 0.026 and r-Spearman = -0.109; p= 0.028 and r-Spearman = -0.107; respectively), indicating that the value of these variables will be lower when the pregnancy is multiple.

**Table 3:** Uterine artery Doppler flowmetric parameters in Saanen goats obtained by non-invasive spectral Doppler ultrasonography during the gestational period.

**Variable**	**GA**	**N**	**Mean**	**SD**	**CI 95% IL**	**CI 95% SL**
RIUT	28	23	0.52	0.07	0.48	0.55
	42	23	0.52	0.07	0.49	0.55
	56	22	0.59	0.07	0.56	0.62
	70	17	0.58	0.08	0.55	0.62
	84	19	0.58	0.05	0.56	0.60
	98	19	0.53	0.08	0.49	0.56
	112	21	0.53	0.09	0.49	0.57
	126	20	0.56	0.07	0.53	0.59
	143	25	0.58	0.09	0.54	0.61
	148	23	0.56	0.09	0.52	0.60
PIUT	28	23	0.80	0.18	0.72	0.87
	42	23	0.78	0.23	0.69	0.87
	56	22	0.98	0.19	0.89	1.10
	70	17	0.97	0.20	0.88	1.06
	84	19	0.95	0.16	0.88	1.02
	98	19	0.82	0.20	0.73	0.91
	112	21	0.81	0.22	0.72	0.91
	126	20	0.85	0.14	0.79	0.91
	143	25	0.91	0.24	0.82	1.01
	148	23	0.87	0.25	0.77	0.97

DA: gestational age; N: number of assessed animals; Mean: average of the values obtained; SD: standard deviation; CI: confidence interval; IL: inferior limit; SL: superior limit; RIUT: uterine artery’s resistance index; PIUT: uterine artery’s pulsatility index.

## Discussion

This study presents important data that contribute to the understanding of the gestational vascular physiology in goats. The uterine artery could be assessed without difficulties throughout the entire pregnancy, while the umbilical artery presented correlation with gestational age.

The resistance index (RI) is one of the most important indices of tissue vascular perfusion due to its negative relationship with vascular perfusion. That is, decreasing resistance increases vascular perfusion and vice versa, just as increases in PI indicate decreases in tissue perfusion (Elmetwally, 2016; Gupta et al., 2009). As pregnancy progresses, PI and RI are expected to progressively decrease, indicating greater fetal blood flow (Elmetwally et al., 2016a). However, in our study, no significant variations of these indices related to the uterine artery were observed. Numerically, there was an increase in the mean RI and PI values between assessments on days 42-84, indicating lower vascular perfusion in this period, followed by a decrease in these values consistent with the increase in physiological blood flow and expected with advancing gestation.

No significant interaction between time of evaluation (60 days, 90 days and 120 days of pregnancy and peripartum) and uterine artery hemodynamic indices (PI and (RI) were also described by Veiga et al. (2018) in ewes. However, they also reported a progressive increase in PSV and time average maximum velocity (TAMAX) of the uterine artery from 60 days until 90 days of pregnancy, when a stabilization in blood flow occurred. These authors suggested that increased uterine blood flow during pregnancy is a consequence of a greater blood flow velocity rather than uterine artery resistance, and that the increase in uterine artery diameter (vasodilation) was not sufficient to change vascular resistance. This finding may explain the absence of a direct correlation between uterine artery indices and gestational age and also in the prepartum period (Figure 3) in our study.

The lack of significant variation in the indices in our study differs from that described by other previous studies in sheep and goats (Beltrame et al., 2017; Elmetwally et al., 2016a). When comparing the RI and PI values in the present study with the aforementioned researches, since the beginning of pregnancy, the values were already lower than those. These differences may indicate that the goats evaluated in this study had a higher mean blood supply, especially due to the high number of twin pregnancies. In humans, it has been reported that uterine arteries exhibited a steady decrease in PI in singleton pregnancies whereas in twin pregnancies, PI exhibited a steady decrease up to 27 weeks' gestation and remained unchanged thereafter (Chen et al., 1998).

It is also worth emphasizing that the mean RI and PI values reported by Beltrame et al. (2017) are similar to those of our study when compared (RI= 0.62; 0.60; 0.58 / PI= 1.15; 1.05; 0.97; in early, medium and late pregnancy, respectively). Another hypothesis is that the location of arterial flow is divergent between the studies, taking in consideration that the maternal-fetal vascularization is formed by closely associated vascular systems, with several branches and anastomoses (Schoenau et al., 2005), which can make it difficult to individualize and standardize. In this sense, there is also the variation related to the Doppler insonation angle used in the different studies, considering that the velocity of blood flow usually changes according to the Doppler angle (Serin et al., 2010).

Doppler ultrasonography is a useful method to assess the effects of maternal anxiety on intrauterine fetal growth in pregnant sheep and goats (Elmetwally et al., 2016b). According to the authors, the uterine artery RI in goats was higher in more anxious/inactive animals between the 8-20 weeks of gestation. In our research, since they are dairy animals, most of the goats were docile and easy to handle, however, some animals had a more aloof temperament as standard behavior. Therefore, this is also a factor that can influence the values obtained in different animals during pregnancy, which may result from the individual temperament of the animal.

The free-floating umbilical cord was first identified on day 28 of pregnancy in our study and on day 39 of pregnancy by Kumar et al. (2015) and the umbilical wave pattern similar to that described by these authors in Beetel goats. However, in our exams, it was only possible to evaluate the cord until day 133 of gestation, a reduced period when compared to Elmetwally and Meinecke-Tillmann (2018), who evaluated the umbilical cord of fetuses of Saanen goats until the 20^th^ week of gestation. This reduction in the evaluation period was due to the difficulty in locating and individualizing the umbilical artery blood flow in the final phase of pregnancy and, even though the umbilical cord was visualized, there was no formation of an adequate spectral tracing that would allow a reliable evaluation.

In our study, a gradual decrease in the RIUMB was observed from day 105 of pregnancy, while the PIUMB increased between days 63-84 and then declined again. In the study by Kumar et al. (2015), the authors found that the PI value increased significantly during 42-48 days of gestation and decreased significantly from day 98 to 105. On the other days of gestation, there was no significant increase or decrease and there was no significant increase or decrease in the RI value, differing from our results.

In our study, the umbilical artery blood flow waveform was characterized only by the systolic waveform up to 84 days of gestation. Later, a diastolic waveform was also detected, like that observed by Serin et al. (2010), also in Saanen goats where the same change in the flow pattern was noticed from the 85th day of gestation. These findings result from physiological changes in blood vessels related to the gestational process. As reported by Reynolds et al. (2005) in sheep, there is an increase in placental vascularization during pregnancy and these are followed by an exponential increase in umbilical blood flow.

Although the umbilical artery Doppler indices were correlated with gestational age in our study, it was not possible to establish predictive equations suitable for practical use, since the coefficients of determination of these presented values below 50%, and coefficient values below 70% are not considered appropriate for estimating gestational age.

As limitations of this study, we emphasize the non-acquisition of Doppler indices from the umbilical artery in the last weeks of pregnancy and in the peripartum period, this failure being attributed to the use of a linear transducer with a frequency of 7.5MHz, which limits the ultrasound depth, being crucial especially in the final third of pregnancy.

## Conclusions

The study of the blood flow of vessels involved in pregnancy in goats with a weekly frequency is important to acquire a more complete picture of the hemodynamic changes resulting from this process. It is expected that the results contribute to a broader understanding of the hemodynamic changes resulting from pregnancy in goats.
